# Is disinformation more likely to spread? A fuzzy-set qualitative comparative analysis of emerging infectious diseases on China’s short video platform

**DOI:** 10.1136/bmjopen-2023-083351

**Published:** 2024-10-21

**Authors:** Yongbin Xu, Sanmei Wen, Liwen Zhang, Jing Su

**Affiliations:** 1 School of Journalism and Communication, Tsinghua University, Beijing, China; 2 College of Arts and Media, Tongji University, Shanghai, China; 3 School of Humanities, Tsinghua University, Beijing, China

**Keywords:** Public health, Social Media, PUBLIC HEALTH, China

## Abstract

**Abstract:**

**Objectives:**

This study aimed to develop a model for the dissemination of information on emerging infectious diseases (EIDs) by identifying the specific features of mpox (monkeypox)-related short video content that encourages public sharing.

**Design:**

This was an exploratory analysis of the dissemination of information on short video platform.

**Main outcome measures:**

Focusing on Douyin (TikTok in mainland China), this study collected data from the top 500 most popular short videos using ‘monkeypox’ as the keyword from 1 May 2022 to 31 October 2022. Under the guidance of the health belief model, the videos were coded using two sets of conditional variables: information type variables and information content variables. The information type variables distinguish between fact-checking information and disinformation. Regarding information content variables, this study integrated the features of audiovisual media with the needs of the Chinese public, introducing efficacy information. The study then used fuzzy-set qualitative comparative analysis to analyse the correlation and consistency between the video contents and the level of public sharing, which was the outcome variable. Subsequently, a Poisson regression model was estimated to verify their significance on video-sharing volume.

**Results:**

The results showed that there were three configurations of short video content related to mpox which could trigger a high level of sharing among the general public. It was found that the number of fact-checked cases in the most widely disseminated short videos of mpox was 21.8:1 compared with the number of disinformation cases. Therefore, it can be concluded that fact-checked information was more likely to spread than disinformation in the case of an outbreak of EIDs on China’s short video platforms. Based on the analysis of the three configurations, we also found that they separately formed three paths of the short video communication model, and each path had a more significant variable playing a central role. We named each pathway after the core variable: authoritative source path, personal efficacy path and disinformation path.

**Conclusions:**

This study developed a model for information dissemination of EIDs and found that fact-checked content was more likely to spread on Chinese short video platforms instead of disinformation. It also explored public demand for guidance on EIDs precautions.

**STRENGTHS AND LIMITATIONS OF THIS STUDY**

- This study focused on the relatively underexplored area of mpox disinformation dissemination in academic research.
- This study conducted a qualitative comparative analysis of misinformation related to emerging infectious diseases (EIDs) on short video platforms to identify which video content promotes public action.
- In this study, we introduced efficacy information and divided it into social efficacy and personal efficacy to address the health belief model deficiency in accounting for the impact of social norms on behaviour.
- The analysis considers the dissemination of disinformation and fact-checked information within the Chinese context, recognising that local characteristics may influence the findings.

## Introduction

The escalating concern about emerging infectious diseases (EIDs) in the risk-aware 21st century is underscored by the outbreaks of diseases such as SARS, H1N1 and COVID-19.^1^,^3^ People are increasingly focusing on the infodemic related to EIDs on social media.^4^,^6^ Concerns about infodemics have focused on managing health-related rumours on social media platforms, while the WHO has woven a call for more active use of social media to disseminate health information, and to counter disinformation about EIDs in particular, to journalists, doctors and the public.^7^ At the beginning of the COVID-19 outbreak, the WHO published a report entitled Dealing with the Infodemic.^8^

The infodemic has garnered attention due to the concern that the accumulation of disinformation during an EID outbreak can have more widespread and faster-reaching impacts than fact-checked information. However, it is worth scrutinising whether this assumption is unchallengeable. This study argues that this requires deeper and more detailed empirical analyses of the media environment and the propensity of audiences to share information in the context of different countries.

China’s media landscape can be roughly divided into mainstream media and social media. Mainstream media typically adopt authoritative information sources for their stories, leading to higher trust among the Chinese public. However, with the rise of the internet and digital technologies, the way people access information has changed. With its immediacy, interactivity and personalisation, social media has attracted a large number of users. According to the China Internet Network Information Center, the number of mobile internet users in China reached 897 million by the end of March 2020.^9^ Statistics show that Chinese internet users not only lead the world in number but also exhibit the highest level of trust in social media platforms.^10^ Social media, nonetheless, is a mix of authoritative platforms involving a variety of Internet celebrities and independent users where gatekeeping is more difficult to do. Despite the prevalence of disinformation, there has not yet been a more detailed study of who the public trusts more in the event of a public epidemic outbreak.

In recent years, with the rise of short video platforms, the viral spread of disinformation has become even more complicated. For instance, following the WHO’s report of international mpox (monkeypox) cases on 7 May 2022, the discussion about the mpox epidemic on Douyin (TikTok in mainland China) was heated. Notably, the prevalence of disinformation concerning the mpox epidemic catalysed public panic and concern in China. Despite the widespread dissemination of disinformation on China’s short video platforms, the literature regarding short video platforms is still lacking due to the high technological threshold of research in comparison to comparatively textual media such as Weibo, Facebook and Twitter. Therefore, it is particularly important to study the dissemination of disinformation in short video platforms in the Chinese context.

The health belief model (HBM) is a theoretical model used to explain and predict health behaviour and is commonly used to study how disinformation on social media affects public behaviour,^11 12^ where questionnaires and qualitative research methods are typically applied.^13^,^15^ Usually, HBM is used in questionnaire studies to measure the relationship between the public’s health beliefs and health behaviours at the time of EIDs. Today, short videos can intervene with people’s psychological activities such as perceptions, attitudes and beliefs through video content, which can change people’s behaviour and encourage the likelihood of sharing and dissemination. Thus, it is necessary to investigate whether the HBM can also be applied to the analysis of the content disseminated in such forms, and which dimensions need to be extended. In this study, we introduce efficacy information and split it into social efficacy and personal efficacy information to compensate for HBM’s lack of information on the impact of social norms on people’s behaviour.^16^ This study uses a combination of qualitative and quantitative approaches to explore the public sharing of mpox-related content on Douyin and discuss whether the dissemination of disinformation has more widespread and faster-reaching impacts than fact-checked information. This research aims to further develop the HBM model regarding EIDs and specifically contributes to the dissemination models of short video platforms.

## Methods

### Study design and data source

The search protocol adheres to stringent guidelines to ensure the comprehensiveness and relevance of the dataset. The keyword ‘monkeypox’ serves as the linchpin for the search, considering its direct alignment with the research focus.

Per the research questions, this study opts to employ fuzzy-set qualitative comparative analysis (fsQCA) for exploration. The study selects the top 500 popular mpox-related short videos on the Douyin platform as the subject of research. The coders are then trained to screen and encode the videos. Subsequently, a qualitative comparative analysis is performed on the video variables.

The study initially sources data from the China short video data platform Douyin (https://www.douyin.com/video), using ‘monkeypox’ as the pivotal keyword.^17^ This strategic approach captured the top 500 most popular mpox-related short videos on Douyin, determined by the number of likes and views, covering the period from 1 May 2022 to 31 October 2022, which corresponds to the first 6 months following the outbreak of the mpox pandemic. Rigorous screening measures are subsequently implemented to filter out content not directly relevant to mpox, leading to the identification of 443 pertinent samples.

### Statistical analysis

**Figure 1:**
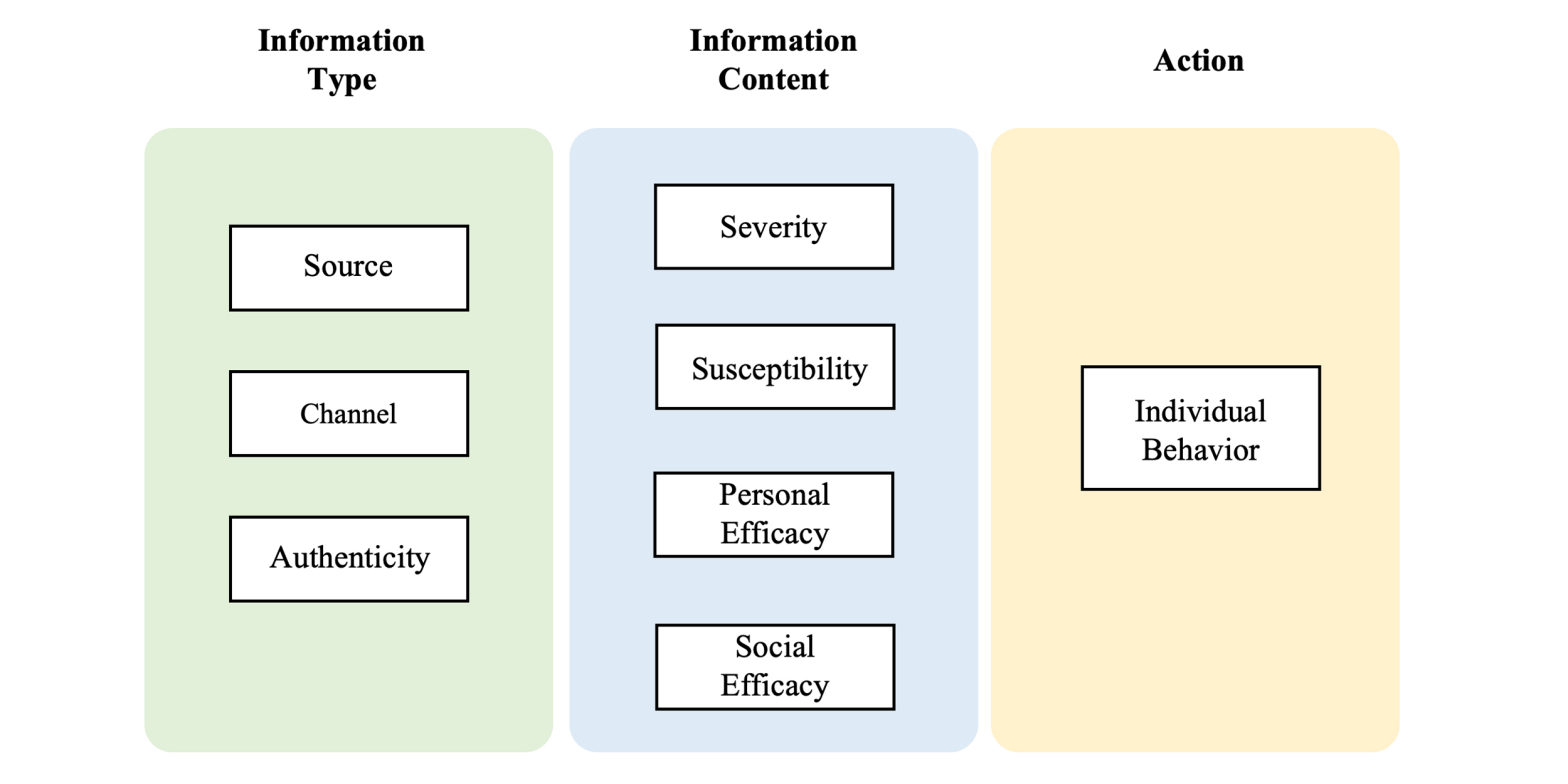
Short video media information communication variables for EIDs. EIDs, emerging infectious diseases.

Following the rigorous selection and screening process, the video content was systematically classified as conditional variables. Under the guidance of HBM, these conditional variables were divided into two categories, information type and content.^18^ As previously mentioned, HBM does not take into account the influence of social norms on the behaviour of the population, and China is a collectivist culture wherein people’s health attitudes and personal behaviour are heavily influenced by social norms.^19^,^21^ In the information content variable, this study considered the context of China and combined the characteristics of audiovisual media and Chinese public demand, introducing efficacy information as the content variable (figure 1). This not only takes into account the personal efficacy information mentioned in traditional research^22^ but also investigates social efficacy in the context of China’s collective culture. Online supplemental table 1 provides comprehensive details on the selection and assignment of the conditional variables.

Data coding was conducted by five postgraduate students with expertise in health communication. Coders received training prior to the formal coding process to ensure the reliability of the final coding scheme. A reliability score of 0.96 was achieved by precoding 10% of the samples. It is pertinent to note that information delivered by the media nearer to the audience tends to attract more attention as per the principle of news proximity. Consequently, social efficacy variables were categorised into Chinese and international social efficacy. Chinese social efficacy was assigned a value of 1, whereas international social efficacy was assigned a value of 0.75. The appendix contains additional information on variable judgement and assignment.

This study analysed the 443 most popular videos related to mpox and used the number of shares as the outcome variable. Existing research, theories and empirical knowledge were consulted, and direct calibration methods were used to convert the data into fuzzy-set membership scores.^23^ This study adopts the calibration criteria and real case scenarios of Peer C. Fiss,^24^ where the calibration criteria for the number of shares is set at the 0.5 percentile, full non-membership is calibrated at the 0.25 percentile, and full membership is calibrated at the 0.75 percentile. Online supplemental table 2 demonstrates the calibration information for the outcome variable.

Finally, given fsQCA’s advantages in handling categorical issues, degree changes and partial memberships,^25^ this study employed fsQCA for exploration. FsQCA was used to calculate the correlation and consistency between the condition variable and the outcome variable. Using fsQCA to derive the necessary conditions or configurations, a Poisson regression model was subsequently employed to verify their significance on video-sharing volume.^26^ This approach facilitates the construction of a short video information dissemination model of EIDs.

### Patient and public involvement

No patients were actively involved in setting the research question, outcome measures nor involved in the design of the study. Patients were not involved in interpretation or write-up of the results, nor are there plans for the results to be disseminated to the patient community affected by this research.

## Results

### Univariate analysis on the promotion of short video sharing

Pertaining to the research question, this study initially investigates whether there exists a singular factor in short video content that promotes public sharing. We first conducted a necessary condition test for the causal factors of conditional variables and outcome variables. Specifically, it examined if a single condition (including its negation) constitutes a necessary condition for a high sharing volume of videos related to mpox. In QCA, a condition is deemed necessary when it is invariably present when the outcome occurs.^23^ Consistency and coverage serve as important criteria for identifying necessary conditions. A condition with its consistency above the typically used threshold (ie, 0.9) and its coverage greater than 0.5 is considered a necessary condition.^27^ Table 1 presents the results of necessary condition tests for videos with high and low sharing volumes related to mpox. The study finds that only the variable of ‘Fact-checked Information’ exceeds a consistency level of 0.9, but its coverage does not exceed 0.5. Thus, there is no singular necessary condition affecting the high sharing volume of mpox videos on the Douyin platform, leading to the conclusion that no single element of short video content promotes public sharing.

**Table 1:** Necessary condition analysis

	High sharing volume		Low sharing volume	
Conditional variables	Consistency	Coverage	Consistency	Coverage
Reliable sources	0.873	0.472	0.891	0.523
Non-reliable sources	0.117	0.499	0.100	0.461
Official channel	0.799	0.478	0.804	0.522
Non-official channel	0.201	0.486	0.196	0.514
Fact-checked information	0.927	0.489	0.894	0.511
Disinformation	0.074	0.391	0.106	0.609
Severity variable	0.875	0.511	0.773	0.489
Non-severity variable	0.125	0.337	0.227	0.663
Susceptibility variable	0.499	0.600	0.307	0.400
Non-susceptibility variable	0.501	0.400	0.693	0.600
Personal efficacy	0.143	0.506	0.129	0.494
Non-personal efficacy	0.857	0.476	0.871	0.524
Social efficacy	0.514	0.501	0.555	0.586
Non-social efficacy	0.576	0.544	0.528	0.541

### Configurational analysis on the promotion of short video sharing

Given the absence of a singular element in short video content that promotes public sharing, this study proceeds to explore from a set-theoretic perspective whether a configuration composed of multiple conditions constitutes a sufficient condition for a high sharing volume of videos related to mpox. Consistency is also used to measure the sufficiency of configurations, but the minimum acceptable standards and calculation methods differ from those for necessary conditions. Based on research by Schneider and Wagemann, the consistency level for determining sufficiency should not be less than 0.75, and the frequency threshold should be determined based on sample size.^28^ Consequently, this study ultimately sets the consistency threshold at 0.75 and the frequency threshold at 1, yielding the following three high-sharing-volume configurations in table 2.

**Table 2:** Configurations sufficient for high sharing volume

	Solutions		
Configuration	1	2	3
Source variable	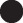	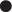	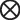
Channel variable	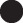	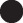	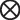
Authenticity variable	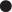	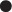	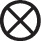
Severity variable	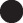		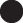
Susceptibility variable	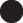	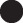	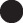
Personal efficacy		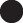	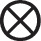
Social efficacy	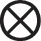	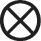	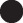
Consistency	0.785	0.815	0.920
Raw coverage	0.186	0.032	0.001
Unique coverage	0.159	0.005	0.01
Overall solution consistency	0.798		
Overall solution coverage	0.201		

Black circles indicate the presence of a condition and circles with ‘X’ indicate its absence. Large circles indicate core conditions; small ones, peripheral conditions. Blank spaces indicate ‘don’t care’

The three configurations presented in table 2 display consistency levels that exceed the minimum acceptable standard of 0.75, both individually and collectively. The overall consistency of these configurations is 0.798, with a collective solution coverage of 0.201. The three configurations can be considered as a sufficient set of conditions for a high sharing volume of mpox videos on the Douyin platform.

Configuration 1 indicates that the presence of variables related to the source, channel, authenticity, severity and susceptibility, along with the absence of social efficacy, contributes to one form of audience sharing. Central to this configuration are the variables related to source, channel, severity, susceptibility and the absence of social efficacy. This configuration has a consistency of 0.785, a unique coverage of 0.159 and a raw coverage of 0.186. This configuration accounts for approximately 18.6% of the mpox short video cases; furthermore, 15.9% of mpox short video cases can only be explained by this configuration.

Configuration 2 suggests another form of audience sharing promoted by the presence of variables related to source, channel, authenticity, susceptibility and personal efficacy, along with the absence of social efficacy. Central to this configuration are the variables related to channel, susceptibility, and personal efficacy, along with the absence of social efficacy. This configuration has a consistency of 0.815, a unique coverage of 0.005 and a raw coverage of 0.032. This configuration accounts for approximately 3.2% of the mpox short video cases.

Configuration 3 demonstrates a third form of audience sharing promoted by the presence of variables related to severity, susceptibility and social efficacy, along with the absence of variables related to source, channel, authenticity and personal efficacy. Central to this configuration are the variables related to severity, susceptibility and social efficacy, along with the absence of authenticity and personal efficacy variables. This configuration has a consistency of 0.920, a unique coverage of 0.01 and a raw coverage of 0.001. This configuration accounts for approximately 1% of the mpox short video cases, and only these cases can be explained by this configuration.

### Comparison of the amount of disinformation and fact-checked information shared

Based on the analysis of the three configurations, configuration 1 and configuration 2 can be used to explain the content from reliable sources and official account channels, and thus they can be classified as the dissemination channels of fact-checked information on Chinese short video platforms. These two configurations explain a total of 21.8% of the mpox short video cases. Configuration 3 helps to explain information from unreliable sources and unofficial accounts, which is classified as a dissemination pathway of disinformation on Chinese short video platforms and explains 1% of the mpox short video cases. Therefore, among the most disseminated short videos related to mpox, the number of videos with fact-checked information is 21.8:1 to the number of videos with disinformation, which concludes that fact-checked videos are not only disseminated more than disinformation but also shared more in the case of an outbreak of EIDs on China’s short video platforms.

### The estimation of the Poisson regression model

After obtaining three high-sharing configurations using fsQCA analysis, this study further employed a Poisson regression model to verify the significant relationship between each variable, these configurations and the sharing volume of mpox videos. The result is presented in table 3. Note that the discussion of sharing volume here uses the raw data of sharing volume.

**Table 3:** The significant relationship between variables, configurations and the sharing volume based on Poisson regression

Variables	Estimate	SE	z-value	P value
Conditional variables				
Source variable	−0.57	0.001	−724.22	<0.001***
Channel variable	0.17	0.001	206.88	<0.001***
Authenticity variable	−0.93	0.001	−626.11	<0.001***
Severity variable	0.68	0.001	724.27	<0.001***
Susceptibility variable	0.55	0.001	1008.66	<0.001***
Personal efficacy	−0.96	0.001	−907.67	<0.001***
Social efficacy	−0.34	0.001	−536.43	<0.001***
Configurations				
Configuration 1	1.22	0.001	1805.8	<0.001***
Configuration 2	−0.78	0.002	−404.1	<0.001***
Configuration 3	0.25	0.001	302.5	<0.001***

Note: *** denotes p<0.001, indicating highly significant results.

First, the Poisson regression results revealed the significant impact of various variables on the dependent variable-video sharing volume. As shown in table 3, the coefficient for the source variable is −0.57, which is statistically significant at the 1% probability level, indicating a significant negative correlation. Additionally, other variables such as the authenticity, personal efficacy and social efficacy variables also have a significant negative impact on the log-expected value of video sharing volume. In contrast, the channel variable, severity variable and susceptibility variable exhibit a significant positive impact on the log-expected value of video sharing volume.

Second, the Poisson regression results demonstrate the significant impact of configurations on video-sharing volume. As shown in table 3, the coefficient for configuration 1 is 1.22, which is statistically significant at the 0.1% probability level. This means that when mpox videos follow the pattern of configuration 1, the log-expected value of video sharing volume increases by 1.22. Similarly, the coefficient for configuration 3 is 0.25, also statistically significant at the 0.1% probability level. This indicates that when mpox videos follow the pattern of configuration 3, the log-expected value of video sharing volume will increase by 0.25.

It is noteworthy that configuration 2 does not exhibit a significantly positive relationship. As shown in table 2, the channel variable, susceptibility variable, and personal efficacy variable play a core role in configuration 2. The channel variable and susceptibility variable show a significant positive impact on video-sharing volume, with coefficients of 0.17 and 0.55, respectively. However, personal efficacy has a significant negative impact on video-sharing volume, with a coefficient of −0.96, which means that when personal efficacy is 1, the log-expected value of video-sharing volume decreases by 0.96. This indicates that personal efficacy has a greater negative impact on the log-expected value of video-sharing volume.

However, due to the small number of videos containing personal efficacy information (only 13.5%), it can only be said that personal efficacy has a greater impact on video-sharing volume, but it cannot directly determine leading to a negative coefficient for configuration 2 in the Poisson regression analysis. Thus, fsQCA is considered an approach that helps bridge the gap when studying complex phenomena. This methodology facilitates the analysis of small samples that may not be representative or of relationships that do not conform to statistical models.^29^ FsQCA can capture units of analysis that traditional variables cannot achieve.^30^ It highlights that even a small number of personal efficacy videos can promote video sharing to some extent.

### Short video information dissemination model of EIDs

By analysing the short video media information communication variables, which are developed by HBM, in these three configurations, this study found that the three configurations form the three paths of the short video communication model, and each path has a more significant variable playing a central role. We used the central variables as the names of these paths. Based on the analysis of the three groups of configurations, it is evident that cases in configurations 1 and 2 explained fact-checked information from reliable sources and official account channels, explaining a total of 21.8% of mpox short video cases. Therefore, they can be classified as communication pathways for fact-checked information on the Chinese short video platform.

Path 1 outlines the factors associated with configuration 1. In this pathway, the variables ‘source’ and ‘channel’ play a central role. When official media releases video content with reliable sources, references to mpox severity and susceptibility, and no mention of social efficacy, it can lead to the emergence of highly shared videos. This pathway shows that the short video on mpox released by official media from reliable sources such as public news reports, government announcements and expert opinion will result in higher sharing behaviour by the public. This pathway is, therefore, called the authoritative source path.

Path 2 outlines the factors involved in configuration 2. In this pathway, the variables ‘susceptibility’ and ‘personal efficacy’ play a central role. When official media releases video content with reliable sources, references to mpox personal efficacy and susceptibility, and no mention of social efficacy, it can lead to the emergence of highly shared videos. This pathway reveals that there is a higher incidence of public sharing behaviour when reliable channels are used to release information, and the content stresses susceptibility and personal efficacy, such as personal protective measures and self-monitoring methods. This pathway is, therefore, called the personal efficacy path.

Configuration 3 involves information released by non-official channels with non-reliable sources, so it can be categorised as the communication pathway to disinformation, explaining a total of 1% of mpox short video cases. In path 3, the set of factors for configuration 3 is presented. In this pathway, when non-official media releases a video with this information from non-reliable sources, and at the same time emphasises the severity and susceptibility of the mpox outbreak, disinformation about social efficacy such as early vaccine preparation in other Western countries and does not mention information about personal efficacy, it results in a highly shared video. This pathway is therefore called the disinformation path.

**Figure 2:**
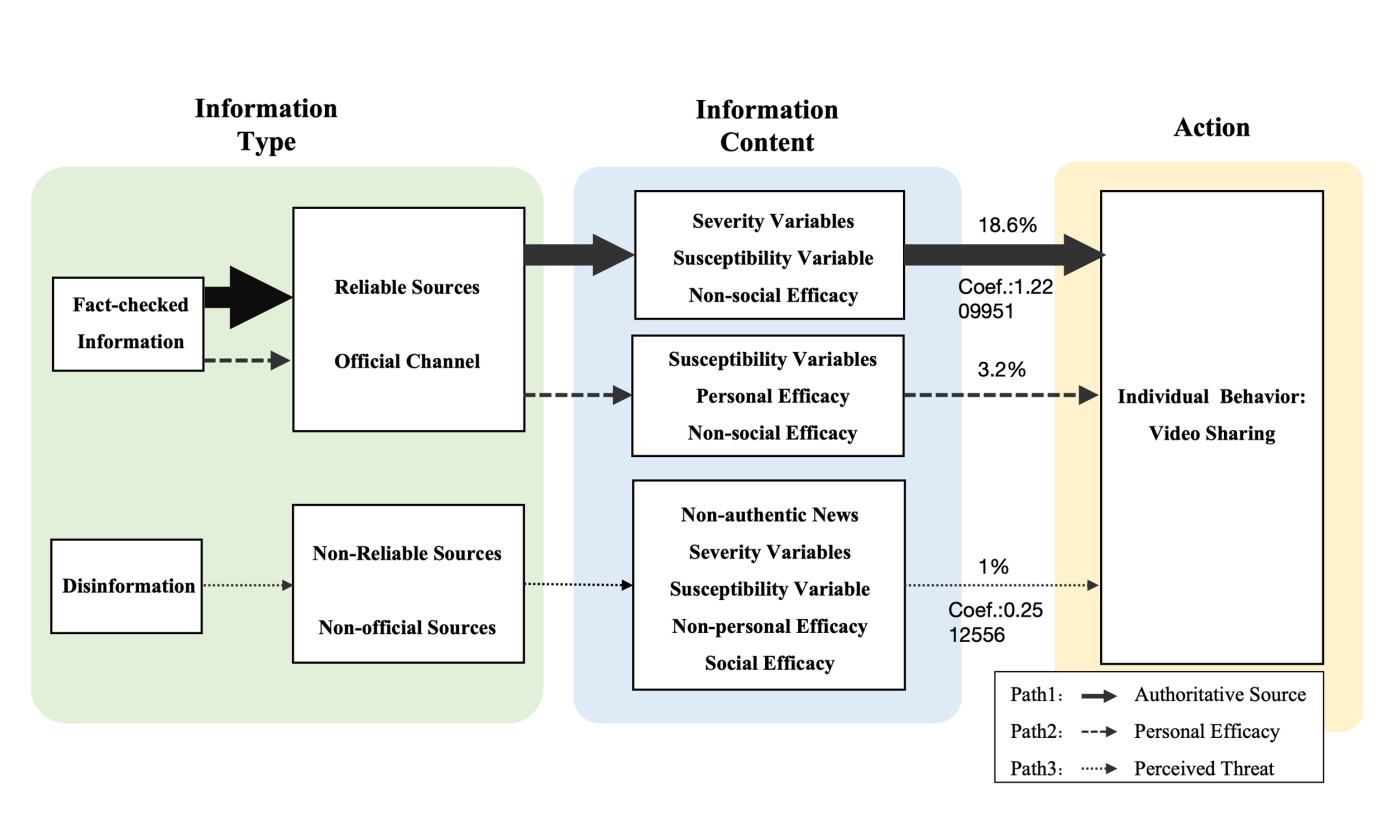
Short video information dissemination model of EIDs.* *Since path 1 can explain about 18.6% of the mpox short video cases, path 2 can explain 3.2% of the short video cases, and about 1% of the mpox short video cases can be explained by this path only, the diameters of the three paths are set in the model in the figure of 18.6:3.2:1.

Therefore, this study forms the EIDs short video information communication model based on the above three paths, as illustrated in figure 2.

## Discussion

### Evaluating the spread of disinformation and fact-checked information on Chinese short video platforms

There is no consensus in previous research on whether the accumulation of disinformation can have more widespread and faster-reaching impacts than fact-checked information. While some studies suggest that disinformation on social media spreads significantly faster, deeper and wider than fact-checked information,^31^ others hold the opposite opinion.^32^ Previous studies of the spread of disinformation on social media during COVID-19 have been conducted in China, Australia, Liberia and Vietnam.^33^,^36^ They focus on the number, themes and characteristics of the spread of disinformation during the epidemic. However, the mechanism of dissemination of disinformation in the Chinese context has not been explored in depth.In this study, we examined the dissemination of disinformation and fact-checked information on Chinese short video platforms, challenging the assumption that disinformation must spread further and faster-reaching than fact-checked information. In the context of China, there are two main reasons why the situation differs from the prevailing assumptions. First, public trust in mainstream media in China is generally high during public health emergencies.^37^ This is perhaps unsurprising, given that social media lacks gatekeepers and is rife with misinformation aimed at gaining web traffic or deceiving users.^38^ Second, we used the HBM to analyse short video content and found that during outbreaks of EIDs, when uncertainty prevails, the public’s understanding and attention to these diseases are likely to align more with the HBM framework’s components of susceptibility, severity and efficacy information. Official mainstream media often emphasise the characteristics of susceptible populations and the measures individuals can take to protect themselves and prevent infection. This focus on efficacy information effectively resonates with the public, greatly encouraging them to share the information. Precisely, the study provided empirical analyses on the spread of disinformation and fact-checked information about EIDs in the context of Chinese short video platforms.

### Three pathways for the dissemination of EIDs information on short video platforms

This study improves the HBM by introducing the concept of efficacy information which is categorised into social efficacy information and personal efficacy information. This study focuses on the impact of specific video content regarding EIDs on the general public at a micro level, develops a model for the dissemination of information based on HBM, and outlines three pathways for short video platforms to promote information as the authoritative source path, personal efficacy path and disinformation path.

The authoritative source path shows that with short videos of mpox released by official media, the public’s sharing behaviour is higher. The personal efficacy path reveals that when reliable channels are used to release information, and the content stresses personal protective measures and self-monitoring methods, there is a higher incidence of public sharing behaviour. Therefore, when a new infectious disease outbreak occurs, authoritative communicators should increase the content of personal efficacy messages on behalf of expert advice. This way, the likelihood of information being disseminated and shared will increase to effectively bridge the differences in risk perceptions that exist between the government, the media and the public,^39^ meanwhile reducing the panic and concern about epidemics caused by EIDs. The disinformation path reveals that when independent media users or unofficial sources spread unsubstantiated information with references to social efficacy messages, there is a significant increase in public sharing behaviour. In summary, the lack or insufficiency of EID-related communication leads to the spread of disinformation, resulting in greater infodemic.

With the above said, an infodemic may not be as frightening as people think, and it is not always the case that an EID outbreak will lead to an infodemic. With the development of the media and the increasing desire of individuals to speak up, there is an inevitable tendency for information abundance during an EID outbreak. But whether a surge in information can be called an infodemic deserves further reflection and clarification. In the case of China, Chinese audiences have been subjected to a long period of acculturation by the mainstream media, which makes them more willing to trust such sources in the event of an outbreak of EIDs. As mainstream media continues to exert its traditional media-era authority over short video platforms^40^ and has its presence embedded within, social media now also allows experts to continuously provide authoritative, fact-checked information.

## Conclusion

Drawing on the HBM, the present study introduces the concept of efficacy information and conducts an empirical investigation on the communication content of EIDs on short video platforms. The study develops the model, with a particular focus on the general public at a micro level. We also summarise three types of high-sharing pathways of current Chinese media EIDs pathways: authoritative source path, personal efficacy path and disinformation path.

The case of China shows that poor prevention and control of outbreaks cannot be blamed on information epidemics for the reasons discussed. The public’s right to know and speak up about infectious diseases must be fully recognised. As a result, the study explores the public’s need for personal prevention guidance on EIDs. Authoritative communicators should not only emphasise risk perception without guiding personal efficacy, which increases social panic,^41^ but also stress the content of social efficacy messages on behalf of the government and personal efficacy messages on behalf of experts.

Given the large number of alternative short video platforms available in China, each targeting a different demographic, future studies should extend the scope to include a horizontal comparison across multiple platforms.This would afford a more comprehensive understanding of public engagement and sentiment towards emerging health threats in China.

## supplementary material

10.1136/bmjopen-2023-083351 online supplemental file 1

## Data Availability

Data are available in a public, open access repository.
