# Supplementary material for: Is disinformation more likely to spread? A fuzzy-set qualitative comparative analysis of emerging infectious diseases on China’s short video platform
Source: BMJ Open. 2024 Oct 21;14(10):e083351. doi: 10.1136/bmjopen-2023-083351 (PMC11499787; doi:10.1136/bmjopen-2023-083351)
Supplement: online supplemental file 1 [file bmjopen-14-10-s001.pdf]

# Supplemental material

**Supplemental Table 1.** Selection and assignment of conditional variables

| Variables | | Variable types | Mesurement | Assign |
|---|---|---|---|---|
| Information Type | Information Source | Reliable Sources | Information claimed to be quoted is from public news reports, government announcements, expert opinions, etc. | 1 |
| | | Non-Reliable Sources | Information not claimed to be quoted from reliable sources | 0 |
| | Channel | Official Channels | Author is accredited as an official media account | 1 |
| | | Non-official Channels | The author is not accredited as an official media account | 0 |
| | Authenticity | Fact-checked Information | Based on scientific evidence or fact-checked information | 1 |
| | | Disnformation | False information that is given deliberately | 0 |
| Information Content | Severity | Severity variable | Description of clinical symptoms, epidemiological situation or diagnostic situation of the disease. | 1 |
| | | Non-severity Variable | No description of the clinical symptoms, epidemiological situation or confirmed diagnosis of the disease. | 0 |
| | Susceptibility | Susceptibility variable | Description of the characteristics of the infected population, the route of infection or susceptible people | 1 |
| | | Non-susceptibility variable | No description of the characteristics of the infected population, routes of infection or susceptible groups | 0 |
| | Personal Efficacy | Personal effectiveness | Description of Physical isolation or reminder of self-monitoring and reporting | 1 |
| | | Non-personal efficacy | No description of physical isolation or reminder of self-monitoring and reporting | 0 |
| | Social Efficacy | Social Efficacy | Chinese Social Efficacy<br>Description of the Chinese release of the Mpox Surveillance, Early Warning and Reporting and Vaccine | 1 |

| | | | |
|---|---|---|---|
| | International Social Efficacy | Description of the international release of the Mpox Surveillance, Early Warning and Reporting and Vaccine | 0.75 |
| | Non-Societal Efficacy | No description of the release of mpox surveillance, early warning and reporting and vaccines | 0 |

**Supplemental Table 2.** Calibration of Outcome Variable

| | Outcome | Calibration | | |
|---|---|---|---|---|
| | | Full Membership | Crossover Point | Full Nonmembership |
| **Outcome Variable** | **Number of video shares** | 18253 | 6371 | 2312.5 |
