# Supplementary material for: Is disinformation more likely to spread? A fuzzy-set qualitative comparative analysis of emerging infectious diseases on China’s short video platform
Source: BMJ Open. 2024 Oct 21;14(10):e083351. doi: 10.1136/bmjopen-2023-083351 (PMC11499787; doi:10.1136/bmjopen-2023-083351)
Supplement: Supplementary file 2 [file bmjopen-2023-083351.reviewer_comments.pdf]

PEER REVIEW HISTORY

BMJ Open publishes all reviews undertaken for accepted manuscripts. Reviewers are asked to complete a checklist review form and are provided with free text boxes to elaborate on their assessment. These free text comments are reproduced below.

ARTICLE DETAILS

**TITLE (PROVISIONAL)**	Is Disinformation More Likely to Spread? A Fuzzy-set Qualitative Comparative Analysis of Emerging Infectious Diseases on China's Short-video Platform
**AUTHORS**	Xu, Yongbin; Wen, Sanmei; Zhang, Liwen; Su, Jing

VERSION 1 - REVIEW

**REVIEWER NAME**	Andersen, Martin
**REVIEWER AFFILIATION**	University of North Carolina at Greensboro Bryan School of Business and Economics, Economics
**REVIEWER CONFLICT OF INTEREST**	None
**DATE REVIEW RETURNED**	08-Feb-2024

**GENERAL COMMENTS**	Summary<br>This article seeks to understand the characteristics of frequently shared short videos related to mpox on Douyin. The authors collected the 500 most shared videos on Douyin that referenced the search term "monkeypox" and analyzed the content of those videos before using a fuzzy set QCA method to identify characteristics that are associated with a high probability of sharing.<br>Positives<br>The study is novel and, as the authors note, it is important to understand how short-format videos (TikTok, Douyin, etc.) can be used to disseminate both factual and non-factual information.<br>Major items<br>1. My biggest concern is with the sampling strategy that the authors employed. By choosing the 500 most shared videos the authors are selecting on the key outcome variable and missing an important dimension by which misinformation may spread—a higher creation rate for false information.<br><br>The basic problem is that there may be a tradeoff between spread/shares and the ease of production. If non-factual videos are easier or faster to create and produced at a higher rate then they may overwhelm the higher sharing rate of factual videos.<br><br>An alternative or supplementary sampling strategy would randomly sample from "monkeypox" related videos that first appeared in a particular week.<br>2. I appreciate the qualitative nature of the authors analysis, but I am concerned that the classification into high and low share groups is somewhat arbitrary. An alternative or supplementary approach would be to estimate a Poisson regression model of the number of shares on the characteristics that the authors identified. I would be particularly interested in a model that enters each of the characteristics separately and one that models the three configurations identified in the analysis.

	Minor items<br>(Line numbers refer to the numbers in the original manuscript)<br>Lines 20-22: fsQCA is a method, not an outcome.<br>Abstract Results: the three paths do not sum to 100%, which may confuse a reader. Could you add an “uncategorized” or “other” category for the balance?<br>Line 33: “today’s risk society”? I find that phrasing confusing. I would also avoid the use of “today” and specify a time period.<br>Lines 41-44: there appears to have been an incomplete edit in these lines, otherwise the first sentence is incomplete.<br>Line 45: I disagree that it is an implicit assumption that a quantum of disinformation spreads further and faster than fact-checked information; I think the concern is the accumulation of disinformation.<br>Lines 51-53: I think some citations for the influence and authority of Chinese media would be helpful here. I don’t think the subsequent citations make the case.<br>Line 61: “Chine” should be “Chinese”?<br>Line 71: “in the Chinese” I don’t think the idea that one needs to study short-video media is specific to the Chinese context.<br><br>While the authors correctly used the search term “monkeypox” when they refer to the virus outside of the context of the search terms they should use “mpox” rather than “monkeypox”.

**REVIEWER NAME**	Kant, Rajni
**REVIEWER AFFILIATION**	Indian Council of Medical Research, Policy and Communications
**REVIEWER CONFLICT OF INTEREST**	None
**DATE REVIEW RETURNED**	24-Mar-2024

**GENERAL COMMENTS**	Line 41 under introduction may be deleted as the same has been explained in next line. Authors have used the Health Belief Model (HBM) to analyse the spread of disinformation and concluded that audience shared the evidenced based information more than the disinformation/misinformation.<br>The reason for the same needs to be explained. It shows the digital/health literacy of the population. How it happened. Is there any such effort to educate the community for getting aware about health related information as in most cases it happens reverse.

VERSION 1 – AUTHOR RESPONSE

Reviewer 1

Comment 1: My biggest concern is with the sampling strategy that the authors employed. By choosing the 500 most shared videos the authors are selecting on the key outcome variable and missing an important dimension by which misinformation may spread—a higher creation rate for false information.

The basic problem is that there may be a tradeoff between spread/shares and the ease of production. If non-factual videos are easier or faster to create and produced at a higher rate then they may overwhelm the higher sharing rate of factual videos.

An alternative or supplementary sampling strategy would randomly sample from “monkeypox” related videos that first appeared in a particular week.

Response 1: Thank you for your valuable comments. Unfortunately, we weren't able to entirely switch to a new round of data sampling for the following three reasons. According to our original sampling method, we selected the 500 most popular mpox-related short videos on the Douyin platform, which is determined by the number of likes and views. The reason for such a way of sampling is that according to the dissemination mechanism of short videos on the Douyin platform, these most-liked videos are more likely to be pushed to a larger audience. Previous studies have also adopted this approach, selecting the most popular videos as their research subjects.[Hong, T. I., Bernstein, S. L., Ramirez, A., Gu, A., Agarwal, A. R., Lutton, D. M., ... & Lutton, D. (2023). Analysis of the Perception and Treatment of Osteoarthritis of the Knee Through Social Media: An Observational Study of the Top 100 Viral TikTok Videos. Cureus, 15(11).][Sun, F., Zheng, S., & Wu, J. (2023). Quality of information in gallstone disease videos on TikTok: cross-sectional study. Journal of Medical Internet Research, 25, e39162.] In these studies, researchers focused their analysis on the most popular or viral TikTok videos within their respective topic areas, using the number of likes or views as a proxy for popularity and reach. Since our research primarily focuses on analyzing the impact of short videos on the public, using this mechanism is relatively reasonable. Since our research primarily focuses on analyzing the impact of short videos on the public, using this mechanism is relatively reasonable.
Secondly, as shown in the attached figure which shows a statistical presentation of our original data, the time distribution of these top 500 videos is somewhat random and covers various periods of the mpox outbreak. This trend in the number of videos also reflects the public's attention triggered by two key dates: the WHO's report of mpox cases outside of endemic regions on May 7, 2022, and the first reported case in China, on September 16, 2022. With that said, we hope our method of selecting samples would eradicate your previous concerns on time sequence and randomization.

Figure. Time distribution chart of the top 500 most liked mpox-related short videos

Lastly, it is unfortunate that videos from 2022 are no longer available due to the data being overwritten, so we weren't able to recollect the data. In future research, we will consider adopting your constructive suggestion to use the sampling method of "first appeared in a particular week" and see if comparative insights emerge.

Comment 2: I appreciate the qualitative nature of the authors' analysis, but I am concerned that the classification into high and low share groups is somewhat arbitrary. An alternative or supplementary approach would be to estimate a Poisson regression model of the number of shares on the characteristics that the authors identified. I would be particularly interested in a model that enters each of the characteristics separately and one that models the three configurations identified in the analysis.
Response 2:
We have now incorporated the Poisson regression model into the results section: The Estimation of Poisson Regression Model (Lines 244 to 266 on page 7). We used the Poisson regression model to validate the significant relationship between the three configurations and the number of video shares. We greatly appreciate your constructive suggestion, as no previous studies have integrated Poisson regression into QCA research for validation, making this approach highly innovative. This also allows QCA research results to be verified using other quantitative methods. Using this method, we can verify the significance between the configurations and share volume, demonstrating the scientific validity of the three configuration results and enhancing the persuasiveness of our EIDs short video information communication model.

Comment 3: Lines 20-22: fsQCA is a method, not an outcome.
Response 3: We have revised the wrong expression on Lines 27-29 in the Abstract.

Comment 4: Abstract Results: the three paths do not sum to 100%, which may confuse a reader.

Could you add an “uncategorized” or “other” category for the balance?
Response 4: Thank you for valuable comments. First, I need to clarify that there is no issue with the number of videos explained by the three configurations. The three configurations account for 18.6%, 3.2%, and 1% of the mpox short video cases, respectively. The remaining video cases are indeed “uncategorized” because their content composition differs from that of the videos within these three configurations and they do not achieve high share volumes. Secondly, to avoid confusing the reader, we have revised the content to reflect that the number of fact-checked cases in the most widely disseminated short videos of mpox is 21.8:1 compared to the number of disinformation cases. (Lines 34-36 in Abstract)

Comment 5: Line 33: “today’s risk society”? I find that phrasing confusing. I would also avoid the use of “today” and specify a time period.
Response 5: Thank you for your valuable comments. We revised “today’s risk society” to “the risk-aware 21st century”. (Lines 62-63 on page 1)

Comment 6: Lines 41-44: there appears to have been an incomplete edit in these lines, otherwise the first sentence is incomplete.
Line 45: I disagree that it is an implicit assumption that a quantum of disinformation spreads further and faster than fact-checked information; I think the concern is the accumulation of disinformation.
Response 6: We revised these lines accordingly. (Lines 71-73 on page 1)

Comment 7: Lines 51-53: I think some citations for the influence and authority of Chinese media would be helpful here. I don’t think the subsequent citations make the case.
Response 7: We refined the text and added citations from the China Internet Network Information Center’s 2020 data on Chinese mobile internet users to illustrate the influence and authority of Chinese media. (Lines 77-85 on page 1)

Comment 8: Line 61: “Chine” should be “Chinese”?
Response 8: We not only revised this term in lines 91-93, page 2, but we also made additional wording changes and performed a thorough double-check on spelling.

Comment 9: Line 71: “in the Chinese” I don’t think the idea that one needs to study short-video media is specific to the Chinese context.
Response 9: We revised the manuscript and refined the text. (Lines 90-100 on page 2)

Comment 10: While the authors correctly used the search term “monkeypox” when they refer to the virus outside of the context of the search terms they should use “mpox” rather than “monkeypox”.
Response 10: Thank you for valuable comments. We have revised “monkeypox” with “mpox” throughout the text, except for search terms.

Reviewer 2
Comment 1: Line 41 under introduction may be deleted as the same has been explained in next line.
Response 2: We revised the manuscript and refined the text. (Lines 71-73 on page 1)

Comment 2: Authors have used the Health Belief Model (HBM) to analyse the spread of disinformation and concluded that audience shared the evidenced based information more than the disinformation/misinformation. The reason for the same needs to be explained. It shows the digital/health literacy of the population. How it happened. Is there any such effort to educate the community for getting aware about health related information as in most cases it happens reverse.
Response 2: Thank you for your valuable comments.
Based on your suggestions, we added an explanation in the “Discussion” section in Lines 319-333 on

page 9, stating that in the context of China, there are two main reasons why the situation differs from prevailing assumptions. One reason is that the public trust in mainstream media during public health emergencies is generally high. Another reason is that we believe digital/health literacy is a fundamental component of people's basic health literacy skills. However, it may not adequately address the public's understanding of and response to emerging infectious diseases during outbreaks characterized by uncertainty. Our study aims to emphasize that, during such periods, the public's understanding and attention to these diseases may align more closely with the Health Belief Model and efficacy information. Mainstream media often highlight the characteristics of susceptible populations and the measures individuals can take to protect themselves and prevent infection. This focus on efficacy information effectively resonates with the public. Therefore, we emphasized this point in our discussion and appreciate your reminder to highlight the importance of health literacy skills.

Requests to add authors to submitted papers
In response to the editor and reviewers' comments, I am writing on behalf of the author team to express our gratitude for the diligent work and valuable feedback provided on our research. Additionally, we wish to request the addition of an author in the third author position to our submitted paper. I would like to provide a thorough rationale for this request to help you understand our motivations.

First, I want to emphasize the substantial contributions made by the new author, Liwen Zhang, to this study. During the revision period, she provided significant methodological support, particularly with the Poisson regression analysis, and assisted with multiple rounds of manuscript editing. Previously, she has been actively involved in our research discussions and data collection efforts. Liwen Zhang's contributions are closely related to the content of the paper. Her involvement has not only enriched the content but also improved the overall quality of the manuscript. We hope you understand our desire to add her as an author and recognize the legitimacy of this decision.

We are willing to provide any additional information or documents to substantiate the new author's contributions and her professional background in the relevant fields. Should you require further information or have any concerns, please do not hesitate to contact me. We greatly appreciate your understanding and support in this matter.

VERSION 2 – REVIEW

**REVIEWER NAME**	Andersen, Martin
**REVIEWER AFFILIATION**	University of North Carolina at Greensboro Bryan School of Business and Economics, Economics
**REVIEWER CONFLICT OF INTEREST**	None to declare
**DATE REVIEW RETURNED**	17-Jun-2024

**GENERAL COMMENTS**	Overall, I am very happy with the revision by the authors. They have largely addressed my comments. I have one major comment on this revision and two minor comments.<br><br>First, does the Poisson model include the eight conditional variables, in addition to the three configurations? I think including the main effects of those conditional variables will resolve the conundrum of the negative coefficient for configuration 2. I would

	like to see this version of the Poisson model, rather than one that ONLY includes the configurations.<br><br>Second, the Poisson coefficients should be presented with two or three, rather than eight, significant digits.<br><br>Third, the paper would benefit from thorough and careful proofreading. For example, "pathway" is misspelled in three locations as "pathay".

VERSION 2 – AUTHOR RESPONSE

Reviewer 1
Comment 1: Does the Poisson model include the eight conditional variables, in addition to the three configurations? I think including the main effects of those conditional variables will resolve the conundrum of the negative coefficient for Configuration 2. I would like to see this version of the Poisson model, rather than one that ONLY includes the configurations.

Response 1: We greatly appreciate your suggestion on utilizing the Poisson regression model, as well as your further guidance on this method. Following your suggestions, we have added the significant impact of each conditional variable on video-sharing volume to the main text (Lines 253-287, pages 7 and 8) and revised Table 3 accordingly (Lines 471-473, page 16). Additionally, we have included relevant literature on Poisson regression in the methodology section (Lines 173, page 4). This has allowed us to conduct a more detailed and comprehensive study and analysis of the data, providing stronger evidence to support the model for information dissemination of EIDs.

Our analysis revealed that personal efficacy has a significant negative impact on video-sharing volume, with a coefficient of -0.96. This suggests that personal efficacy significantly reduces the log-expected value of video-sharing volume. This finding also helps explain the reason for the negative coefficient for Configuration 2.

By combining fsQCA with the Poisson regression method, we were able to scientifically analyze and verify the specific impacts of various variables and the three configurations on video-sharing volume. Once again, we are grateful for your suggestion on utilizing the Poisson regression model.

Comment 2: The Poisson coefficients should be presented with two or three, rather than eight, significant digits.

Response 2: Thank you for your valuable comments. We have adjusted the significant digits of the Poisson coefficients. (Lines 269, 271, 272, 274 on pages 7, 8, and Table 3)

Comment 3: The paper would benefit from thorough and careful proofreading. For example, “pathway” is misspelled in three locations as “pathay”.

Response 3: Thank you for your valuable comments. Each author, including the native speaker LWZ, has now carefully proofread the article multiple times. We have focused on refining the abstract, ensuring consistent use of symbols and acronyms, polishing the language, and unifying tenses in an effort to optimize the manuscript.

Thank you once again for your invaluable feedback and guidance.

VERSION 3 – REVIEW

**REVIEWER NAME**	Andersen, Martin
**REVIEWER AFFILIATION**	University of North Carolina at Greensboro Bryan School of Business and Economics, Economics
**REVIEWER CONFLICT OF INTEREST**	None to declare
**DATE REVIEW RETURNED**	05-Aug-2024

**GENERAL COMMENTS**	I appreciate the authors' work in responding to my comments and I am satisfied with their revision.

VERSION 3 – AUTHOR RESPONSE
